# Three new species of the subgenus *Otocepheus* (*Acrotocepheus*) (Acari, Oribatida, Otocepheidae) from China

**DOI:** 10.3897/zookeys.934.49862

**Published:** 2020-05-19

**Authors:** Li-Hao Zheng, Jun Chen

**Affiliations:** 1 Key Laboratory of Zoological Systematics and Evolution, Institute of Zoology, Chinese Academy of Sciences, Beijing 100080, China Institute of Zoology, Chinese Academy of Sciences Beijing China; 2 College of Life Sciences, University of Chinese Academy of Sciences, Beijing, 100049, China University of Chinese Academy of Sciences Beijing China; 3 Guang’an Vocational and Technical College, Guang’an, 638000, China Guang’an Vocational and Technical College Guang’an China

**Keywords:** oribatid mites, new species, new record, taxonomy, key

## Abstract

Three new species of subgenus Otocepheus (Acrotocepheus): O. (A.) digitatus**sp. nov.**, O. (A.) multigranulatus**sp. nov.**, and O. (A.) occultatus**sp. nov.** are proposed and described based on adult material collected from China, and O. (A.) duplicornutus Aoki, 1965 is reported in China for the first time. A key to Chinese species of the subgenus Acrotocepheus is provided.

## Introduction

The oribatid mite genus *Otocepheus* was first proposed by Berlese (1905) as a subgenus of *Carobodes* with two new species from Java, Carabodes (Otocepheus) longior and C. (O.) crinitus. Later, Berlese (1913) described another new species, *Otocepheus damoeoides*, and a variety, O. longior var. minor, both from Java, which suggested that the subgenus was promoted to the generic rank. Trägårdh (1931) recognized the generic status of the genus *Otocepheus* and selected Carabodes (Otocepheus) longior Berlese, 1905 as the type species for the genus. At present, *Otocepheus* has three subgenera, Otocepheus (Otocepheus) Berlese, 1905, Otocepheus (Aceotocepheus) Aoki, 1965, and Otocepheus (Hexatocepheus) Wen, 1993, and comprises 53 species (Subías 2004, 2020).

The subgenus Otocepheus (Acrotocepheus) was proposed by Aoki (1965), with O. (A.) quateorum Aoki, 1965 as type. The subgeneric characters of Otocepheus (Acrotocepheus) were summarized by Aoki (1965), and identification keys to species from some regions and countries were presented by Aoki (1965) and Corpuz-Raros (2007).

Chen et al. (1992) recorded O. (A.) gracilis (Aoki, 1973) from Anhui, China, which was the first report of genus *Otocepheus* in China. The following year, Wen (1993) proposed the subgenus Otocepheus (Hexatocepheus) with O. (Hexatocepheus) emeiensis from Sichuan, China as type. Until now, only two subgenera and two species of *Otocepheus* were recorded in China (Chen et al. 2010). During studies of oribatid mites from China, we discovered three new species of subgenus Otocepheus (Acrotocepheus)–O. (A.) digitatus sp. nov., O. (A.) multigranulatus sp. nov., and O. (A.) occultatus sp. nov.–and the first record in China of O. (A.) duplicornutus Aoki, 1965. All four of these species are described, and an identification key for all known species of this subgenus in China is provided.

## Materials and methods

The collection locality and habitat for each new species are given in the “Material examined”.

Specimens were mounted in lactic acid on temporary cavity slides for measurement and illustration, except one specimen of O. (A.) digitatus sp. nov., which was mounted on a permanent slide with Hoyer’s medium. The body length was measured in lateral view, from the tip of the rostrum to the posterior edge of the ventral plate. Notogastral width refers to the maximum width in dorsal aspect. Lengths of body setae were measured in lateral aspect. All body measurements are presented in micrometers. Formulas for leg setation are given in parentheses according to the sequence trochanter-femur-genu-tibia-tarsus (famulus included). Formulas for leg solenidia are given in square brackets according to the sequence genu-tibia-tarsus.

General terminology used in this paper follows that of Grandjean (1934), Aoki (1965, 1967), Norton (1977), and Norton and Behan-Pelletier (2009).

### Abbreviations and notations

***Prodorsum***: *ro*, *le*, *in*, *bs*, *ex* – rostral, lamellar, interlamellar, bothridial, and exobothridial setae, respectively; *cos* – costula; *tu* – tutorium; *spa.l* – lamelliform expansion; *tbd*, *tbv* – dorsal and ventral bothridial plate, respectively; *cpm*, *cpl* – medial and lateral prodorsal condyles, respectively; *cex* – extral condyles.

***Notogaster***: *c*, *la*, *lm*, *lp*, *h*-row, *p*-row – notogastral setae; *cnm*, *cnl* – medial and lateral notogastral condyles, respectively; *vm* – vitta marginalis; *ia*, *im*, *ip* – anterior, middle, posterior lyrifissures, respectively; *ih*, *ips* – same, associated with setal rows *h* and *p*, respectively; *gla* – opisthonotal gland opening.

***Coxisternum and lateral podosoma***: *1a*, *1b*, *1c*, *2a*, *3a*, *3b*, *3c*, *4a*, *4b*, *4c* – setae of epimeres I–IV; *met* – mentotectum; *cst* – carina sterinalis; *ap1* – apodeme I; *bo1* – epimeral border I; *Pd I*, *Pd II* – pedotectum I, II respectively; *spd* – sub pedotectum; *fep* – epimeral foramen; *dis* – discidium; *opp* – postpodosomal ornamentation.

***Anogenital region***: *g*, *ag*, *an*, *ad* – genital, aggenital, anal and adanal setae, respectively; *vr* – ventral ridge; *iag*, *iad* – aggenital, adanal lyrifissure respectively.

***Gnathosoma***: *lir* – lower lip ridge of mentum; *a*, *m* – anterior, middle seta of gena; *h* – hypostomal seta of mentum; *v*, *l*, *d*, *cm*, *acm*, *ul*, *su*, *vt*, *lt*, *sup*, *inf* – palp setae; *ω* – palp tarsal solenidion; *ep* – postpalpal seta; *cha*, *chb* – cheliceral setae; *cht* – tooth on dorsal chelicerae; *rbr* – rutellar brush; *ru* – rutellum; *Tg* – Trägårdh’s organ.

***Legs***: *σ*, *φ*, *ω* – solenidia of genu, tibia and tarsus, respectively; *ɛ* – famulus of tarsus I; *d*, *l*, *v* – dorsal, lateral, ventral setae, respectively; *ev*, *bv* – basal trochanteral setae; *ft*, *tc*, *it*, *p*, *u*, *a*, *s*, *pv* – tarsal setae; *Tr*, *Fe*, *Ge*, *Ti*, *Ta* – trochanter, femur, genu, tibia, tarsus of legs, respectively.

## Descriptions

### Otocepheus (Acrotocepheus) digitatus sp. nov.

Taxon classificationAnimaliaSarcoptiformesOtocepheidae

893E8DD3-3748-5F89-B79B-1C151356BCFA

http://zoobank.org/BA165CB6-E780-4F37-942E-F7A36B42F2AE

Figs 1–4 , 5, 6 , 7

#### Diagnosis.

Body size: 1020 × 330. Body surface densely foveolate. Bothridial setae with a long fusiform head and a strongly curved peduncle. Lateral notogastral condyles finger-shaped, with wide base. Notogastral setae different in length, setae *lm*, *lp*, *h*_1_, *h*_2_, *p*_2_ flagelliform and distinctly longer than the others. Genital plates each with two longitudinal, slant furrows.

**Figures 1–4.**
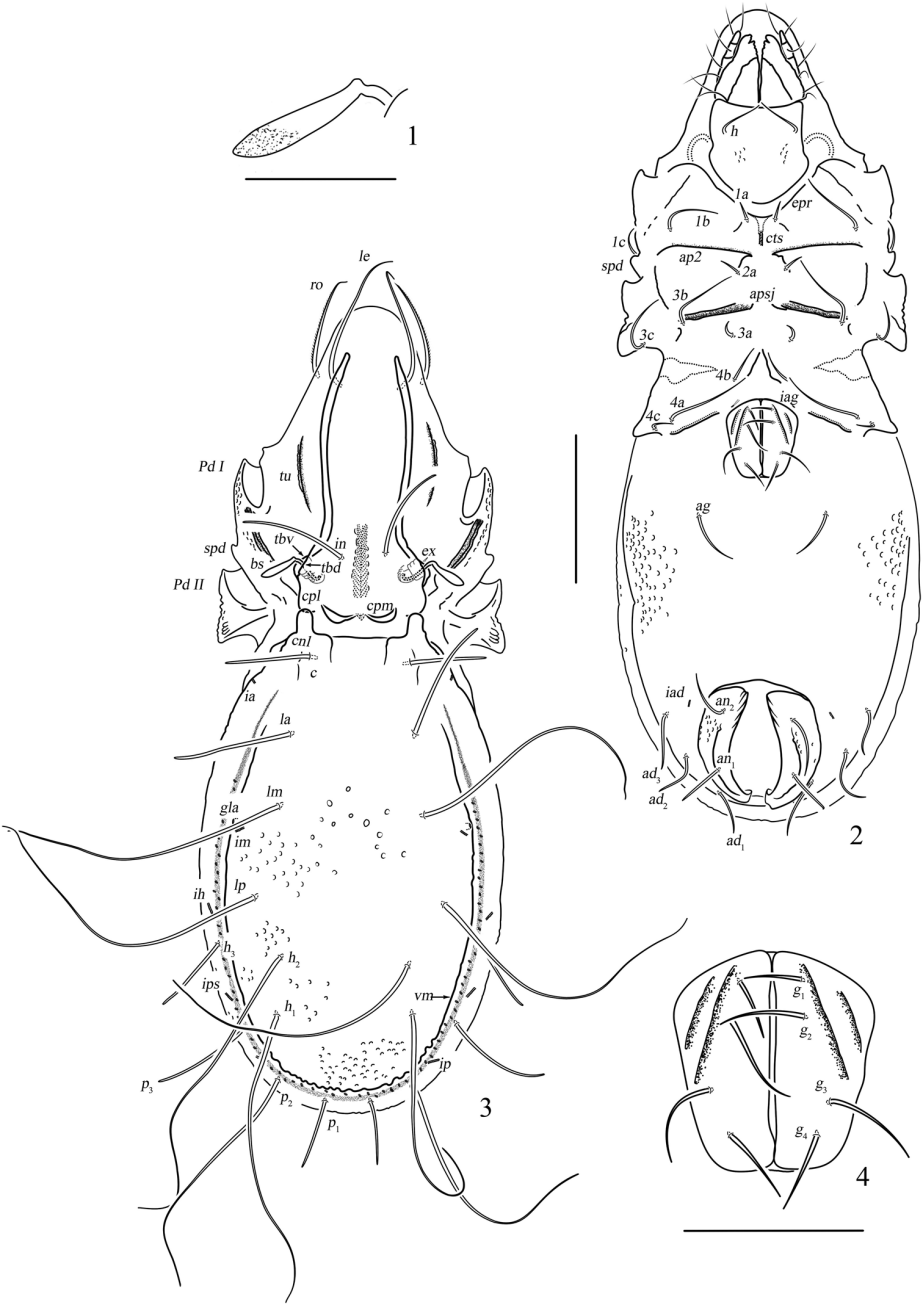
Otocepheus (Acrotocepheus) digitatus sp. nov., adult **1** bothridial setae **2** ventral view (legs not shown) **3** dorsal view **4** genital plate. Scale bars: 100 µm (**1, 4**), 200 µm (**2, 3**).

**Figures 5, 6.**
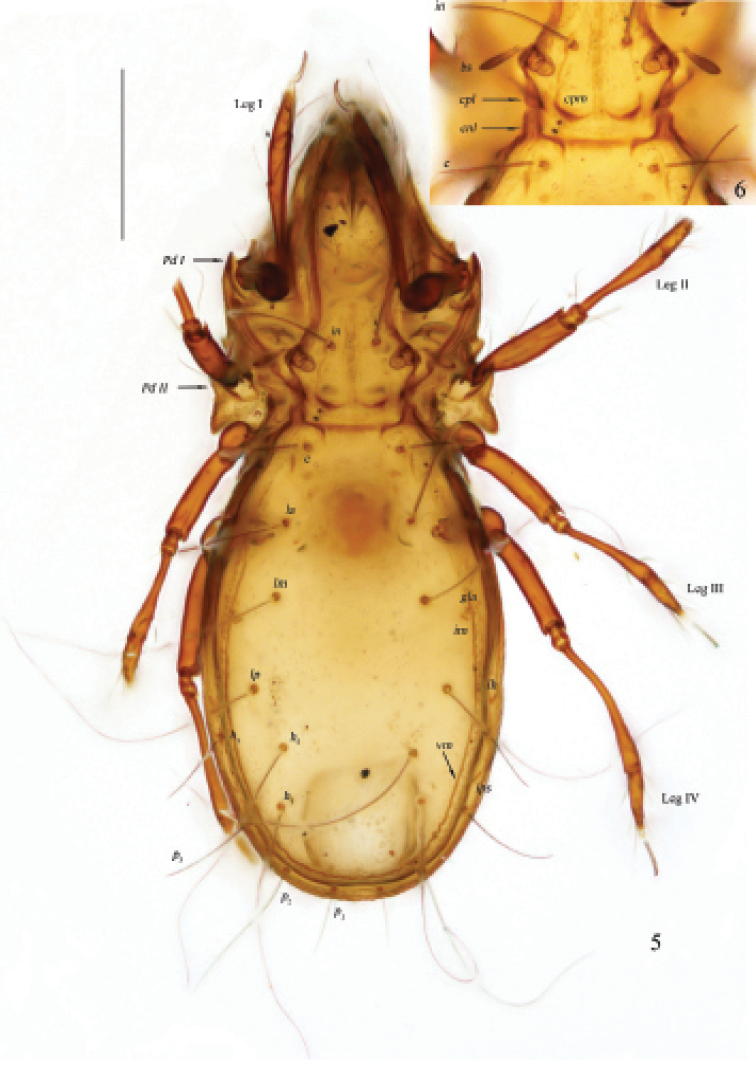
Otocepheus (Acrotocepheus) digitatus sp. nov., adult, microscope images **5** dorsal view **6** prodorsal and notogastral condyles, interlamellar and bothridial setae, notogastral setae *c.* Scale bar: 200 µm (**5**).

**Figure 7.**
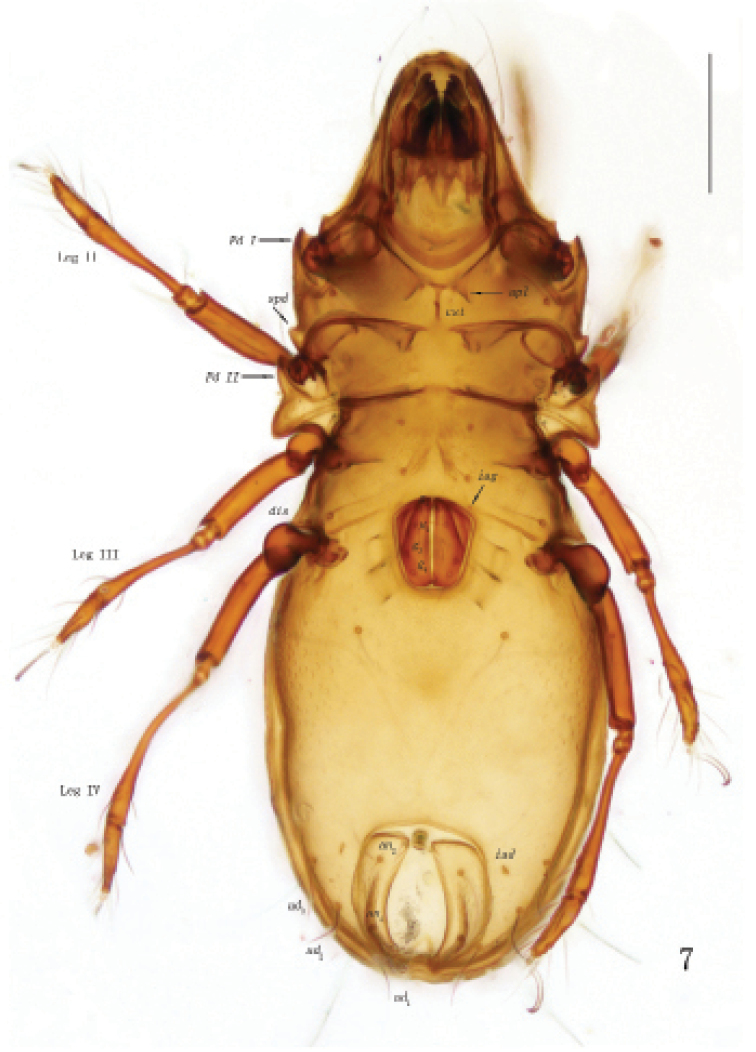
Otocepheus (Acrotocepheus) digitatus sp. nov., adult, microscope image, ventral view. Scale bar: 200 µm.

#### Description.

***Measurements*** (holotype: male). Body length: 1020, notogaster width: 330. Setae length and mutual distance: *bs* 100, *in* 140, *le*180, *ro* 160, *ex* 25; *c*, *la*, *h*_3_, *p*_1_, *p*_3_ range 100–160; *lm*, *lp*, *h*_1_, *h*_2_, *p*_2_ range 300–450; *c*–*c* 120, *la*–*la* 160, *lm*–*lm* 190, *lp*–*lp* 240, *h*_2_–*h*_2_ 160, *h*_1_–*h*_1_ 170.

***Integument*.** Body color light yellow-brownish. Body surface densely foveolate.

***Prodorsum*.** Rostrum broadly rounded. Rostral seta moderately curved inward, densely barbed outside. Lamellar seta inserted behind tip of costula, curved inward, roughened externally. Interlamellar seta barbed, blunt at tip. Bothridial seta with a long fusiform head and a strongly curved peduncle. Exobothridial seta short. Bothridium opening laterally, dorsal bothridial plate straight or curved outward slightly, ventral bothridial plate broadly triangular in dorsal view. Tutorium well developed. Two pairs of prodorsal condyles present, lateral prodorsal condyles broadly rounded, median prodorsal condyles large and rounded, well separated from each other. Mutual distance between ventral bothridial plates nearly equal with that between lateral prodorsal condyles. Subpedotectum well developed.

***Notogaster*.** L/W of notogaster about 1.8. Surface of notogaster densely punctate. Anterior margin of notogater strait. Lateral notogastral condyles finger-shaped, with wide base. Median notogastral condyles absent. Ten pairs of notogastral setae glabrous, setae *c*, *la*, *h*_3_, *p*_1_, *p*_3_ setiform and relatively short in length, while the rest notogastral setae *lm*, *lp*, *h*_1_, *h*_2_, *p*_2_ flagelliform and longer in length. Setae *c*, *la*, *lm* nearly located on the same line. All lyrifissures well visible, *ip* located between setae *p*_2_ and *p*_3_, *ips* between setae *h*_3_ and *p*_3_. Opisthonotal gland opening located anterior and very close to lyrifissure *im.* Vitta marginalis distinct.

***Epimeral and lateral podosomal regions*.** Apodemes I, II and sejugal well developed, apodeme III invisible. Carina sterinalis well developed. Epimeral setal formula 3-1-3-3. Seta *4a* inserted between *4b* and *4c*, and closer to *4c*. Epimeral setae *1b*, *1c*, *3b*, *3c*, *4a* distinctly longer than the rest. Postpodosomal ornamentation invisible.

***Anogenital region.*** Genital plates each with 2 longitudinal, slant furrows. Four pairs of genital setae (mutual distances *g*_1_–*g*_1_≈*g*_2_–*g*_2_≈*g*_4_–*g*_4_<*g*_3_–*g*_3_). Aggenital lyrifissure located close and anterolateral to genital aperture. One pair of aggenital, two pairs of anal (mutual distances *an*_1_–*an*_1_<*an*_2_–*an*_2_) and three pairs of adanal setae similar in length. Setae *ad*_3_–*ad*_3_ below level of anterior margin of anal opening. Adanal lyrifissure located in diagonal position and close to anal aperture, below level of anterior margin of anal opening.

***Legs*.** Monodactylous. Claw of each leg strong and smooth. Formulae of leg setation and solenidia: I (1-4-3-4-16) [1-2-2], II (1-4-3-3-15) [1-1-2], III (2-3-1-2-15) [1-1-0], IV (1-2-2-2-12) [0-1-0]. Leg seta *u* setiform (L-type) on tarsi I, thorn-like (S-type) on tarsi II–IV. Homology of setae and solenidia indicated in Table 1.

**Table 1.** Leg setation and solenidia of adult Otocepheus (Acrotocepheus) digitatus sp. nov., Otocepheus (Acrotocepheus) multigranulatus sp. nov., Otocepheus (Acrotocepheus) occultatus sp. nov., and Otocepheus (Acrotocepheus) duplicornutus Aoki, 1965.

**Leg**	**Tr**	**Fe**	**Ge**	**Ti**	**Ta**
I	*v*'	*d*, (*l*), *bv*"	(*l*), *v*', *σ*	(*l*), (*v*), *φ*_1_, *φ*_2_	(*ft*), (*tc*), (*it*), (*p*), (*u*), (*a*), *s*, (*pv*), *ε*, *ω*_1_, *ω*_2_
II	*v*'	*d*, (*l*), *bv*"	(*l*), *v*', *σ*	*l*', (*v*), *φ*	(*ft*), (*tc*), (*it*), (*p*), (*u*), (*a*), *s*, (*pv*), *ω*_1_, *ω*_2_
III	*v*', *l*'	*d*, *l*', *ev*'	*l*', *σ*	(*v*), *φ*	(*ft*), (*tc*), (*it*), (*p*), (*u*), (*a*), *s*, (*pv*)
IV	*v*'	*d*, *ev*'	*d*, *l*'	(*v*), *φ*	*ft*", (*tc*), (*p*), (*u*), (*a*), *s*, (*pv*)

Note: Roman letters refer to normal setae, Greek letters to solenidia (except *ɛ* = famulus). Single prime (') marks setae on the anterior and double prime (") setae on the posterior side of a given leg segment. Parentheses refer to a pair of setae.

#### Material examined.

Holotype (male, LD-07-117): China, Hainan Province, Qiongzhong City, Limu Mountain, 19°6'18"N, 109°26'42"E, 616 m a.s.l., in soil and debris under reeds, 20 July 2007, collected by Dong Liu.

**Type deposition.** The holotype is deposited in the collection of the Zoological Museum of China, Institute of Zoology, Chinese Academy of Sciences, Beijing (IZAS) (Zhang 2018).

#### Etymology.

The specific name “*digitatus*” is from Latin for “finger-like” refers to the finger-shaped lateral notogastral condyles.

#### Remarks.

The new species is similar to O. (A.) duplicornutus discrepans (Balogh & Mahunka, 1967) from Vietnam and O. (A.) bajau Mahunka, 2000 from Malaysia in having similar shape of lateral notogastral condyles. However, it differs from O. (A.) duplicornutus discrepans by the flagelliform setae *lm*, *lp*, *h*_1_, *h*_2_, *p*_2_ (versus blunt at tips), different length in notogastral setae (versus nearly same length), postpodosomal ornamentation invisible (versus markedly developed), bothridial setae with a long fusiform head (versus flattened distally); it differs from O. (A.) bajau by the flagelliform setae *lm*, *lp*, *h*_1_, *h*_2_, *p*_2_ (versus setiform), setae *c*, *la*, *h*_3_, *p*_1_, *p*_3_ shorter than the other notogastral setae (versus length increasing toward the posterior part of notogaster), lyrifissure *ips* located between *h*_3_ and *p*_3_ (versus between *p*_3_ and *p*_2_), surface of notogaster densely foveolate (versus granulate).

### Otocepheus (Acrotocepheus) multigranulatus sp. nov.

Taxon classificationAnimaliaSarcoptiformesOtocepheidae

90310F78-9619-5B20-8DE4-25957FF9AE29

http://zoobank.org/72F9B5C4-CEBC-49DC-9EFD-2D0D053C6CFA

Figs 8–12 , 13–15 , 16–18 , 19–22

#### Diagnosis.

Body size (*n* = 3): 1150–1240 × 530–609. Bothridial setae with a fusiform head. Lateral notogastral condyles wide, like staircase with two to three layers mainly. An extra pair of condyles (*cex*) located posterior to lateral prodorsal condyles, covered by lateral notogastral condyles. Bothridial seta with a fusiform head. Body surface densely granulate.

**Figures 8–12.**
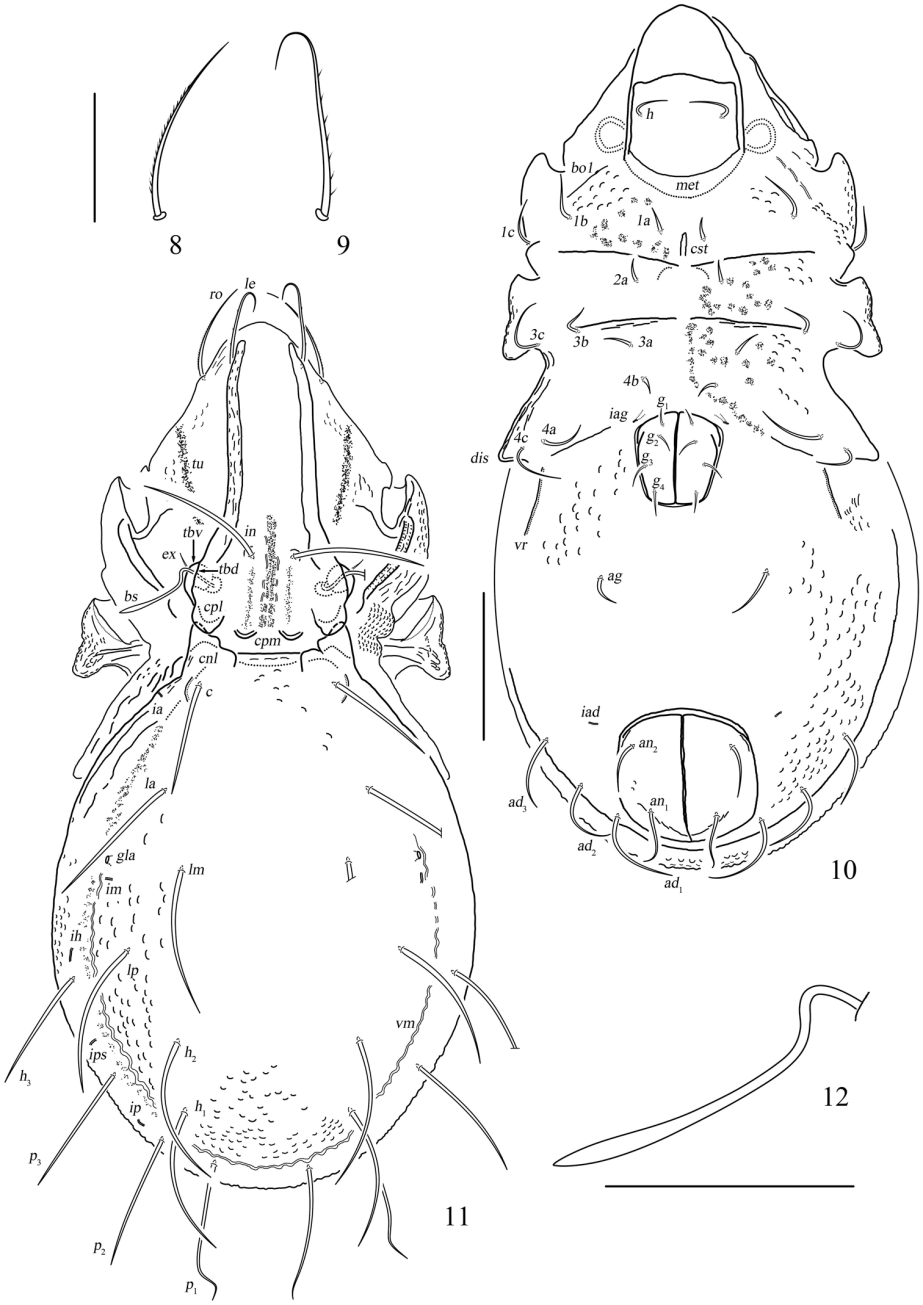
Otocepheus (Acrotocepheus) multigranulatus sp. nov., adult **8** seta *ro***9** seta *le***10** ventral view (legs not shown) **11** dorsal view **12** bothridial seta. Scale bars: 100 µm (**8, 9, 12**), 200 µm (**10, 11**).

#### Description.

***Measurements*.** Body length: 1240 (holotype: female), 1150–1230 (two paratypes: all males); notogaster width: 609 (holotype), 530–600 (paratypes). Setae length and mutual distance (holotype): *bs* 160, *in* 200, *le* 210, *ro* 160, *ex* 35; *c*, *la*, *lm*, *lp*, *h*_1_, *h*_2_, *h*_3_, *p*_1_, *p*_2_, *p*_3_ range 170–200; *c*–*c* 199, *la*–*la* 290, *lm*–*lm* 240, *lp*–*lp* 390, *h*_2_–*h*_2_ 260, *h*_1_–*h*_1_ 240.

***Integument*.** Body color light yellow-brownish. Body surface densely granulate.

***Prodorsum*.** Rostrum rounded. Rostral seta moderately curved inward, densely barbed outside. Lamellar seta inserted behind tip of costula, curved inward, roughened outside. Interlamellar seta barbed, setiform. Bothridial seta with a fusiform head. Exobothridial seta short. Bothridium opening laterally, dorsal bothridial plate straight, ventral bothridial plate broadly rounded in dorsal view. Tutorium well developed. Two pairs of prodorsal condyles present, lateral prodorsal condyles earlobe-shaped, median prodorsal condyles rounded, well separated from each other. An extra pair of condyles located posterior to lateral prodorsal condyles, covered by lateral notogastral condyles. Mutual distance between ventral bothridial plates larger than that between lateral prodorsal condyles. Subpedotectum well developed.

**Figures 13–15.**
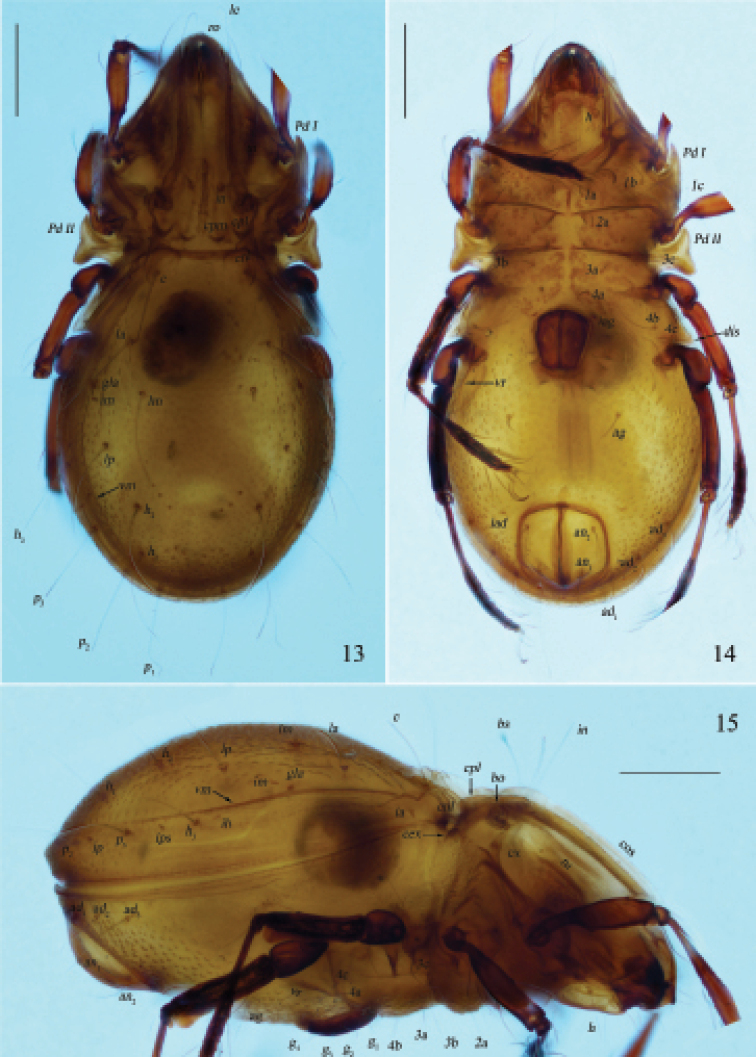
Otocepheus (Acrotocepheus) multigranulatus sp. nov., adult, microscope images **13** dorsal view **14** ventral view **15** lateral view. Scale bars: 200 µm.

***Notogaster*.** L/W of notogaster about 1.2. Surface of notogaster densely and obviously granulate. Anterior margin of notogaster straight. The largest width of notogaster medially, near level of seta *lp.* Lateral notogastral condyles wide, like a lateral view of staircase with two to three steps mainly. Median notogastral condyles absent. Ten pairs of notogastral setae nearly same in length, barbed. A faint, short ridge present lateral to insertion of seta *c.* Mutual distance between setae *p_1_* lager than that between *p_1_* and *p_2_*. Seta *lm* and lyriffissure *im* nearly on same line. Five pairs of lyrifissures visible, *ip* located between setae *p*_2_ and *p*_3_, *ips* between setae *h*_3_ and *p*_3_. Opisthonotal gland opening located anterior to lyriffissure *im.* Vitta marginalis well developed, fainted anteriorly.

**Figures 16–18.**
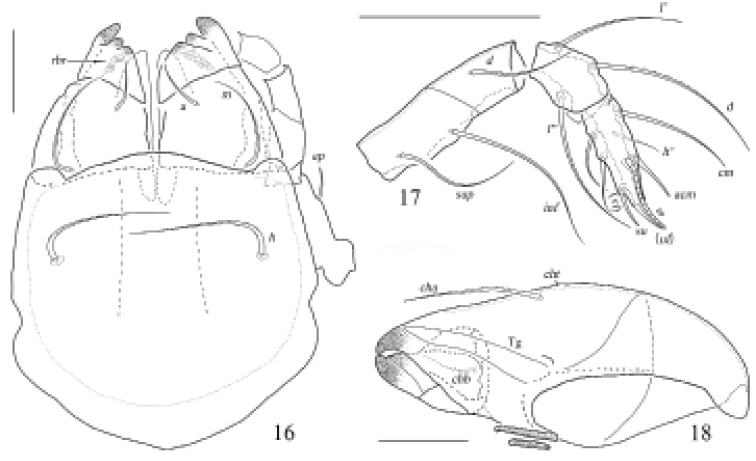
Otocepheus (Acrotocepheus) multigranulatus sp. nov., adult **16** subcapitulum, ventral view **17** right palp (without trochanter), abaxial view **18** right chelicera, adaxial view. Scale bars: 50 μm.

***Epimeral and lateral podosomal regions*.** Apodemes I, II and sejugal well developed, apodeme III invisible. Carina sterinalis well developed. Epimeral setal formula 3-1-3-3. Seta *4a* inserted between *4b* and *4c*, and closer to *4c*. Postpodosomal ornamentation invisible.

***Anogenital region*.** A pair of longitudinal ridges posterior to epimeral seta *4a*. Genital plates smooth. Four pairs of genital setae (mutual distances *g*_1_–*g*_1_≈*g*_2_–*g*_2_<*g*_4_–*g*_4_<*g*_3_–*g*_3_). Aggenital lyrifissure located close and anterolateral to genital aperture. One pair of aggenital, two pairs of anal (mutual distances *an*_1_–*an*_1_<*an*_2_–*an*_2_) and three pairs of adanal setae. Anal and adanal setae barbed like notogastral setae. Setae *ad*_3_–*ad*_3_ below level of anterior margin of anal opening. Adanal lyrifissure situated anterior to level of seta *ad*_3_.

***Gnathosoma*.** Subcapitular setae fistulous, barbed. Adoral setae and their alveoli absent. Rutellum pantelobasic, with typical dentition and rutellar brush. Chelicera chelate-dentate; with a minute denticle proximal to seta *cha*; *cha* longer than *chb*, both of them setiform, barbed; Trägårdh’s organ narrowly triangular. Palp with usual setal formula: 0–2–1–3–8 (+*ω*); setae of *femur* to tibia barbed. Tarsus with four short, blunt distal eupathidia–*acm*, *su*, (*ul*); other tarsal setae smooth or with sparse, inconspicuous barbs; solenidion *ω* connected with seta *ul*’, seta *ul*” medioanteriorly. Postpalpal seta erect, smooth.

***Legs*.** Monodactylous. Claw of each leg strong and smooth. Formulae of leg setation and solenidia: I (1-4-3-4-16) [1-2-2], II (1-4-3-3-15) [1-1-2], III (2-3-1-2-15) [1-1-0], IV (1-2-2-2-12) [0-1-0]. Leg seta *u* setiform (L-type) on tarsi I, thorn-like (S-type) on tarsi II–IV. Homology of setae and solenidia indicated in Table 1.

**Figures 19–22.**
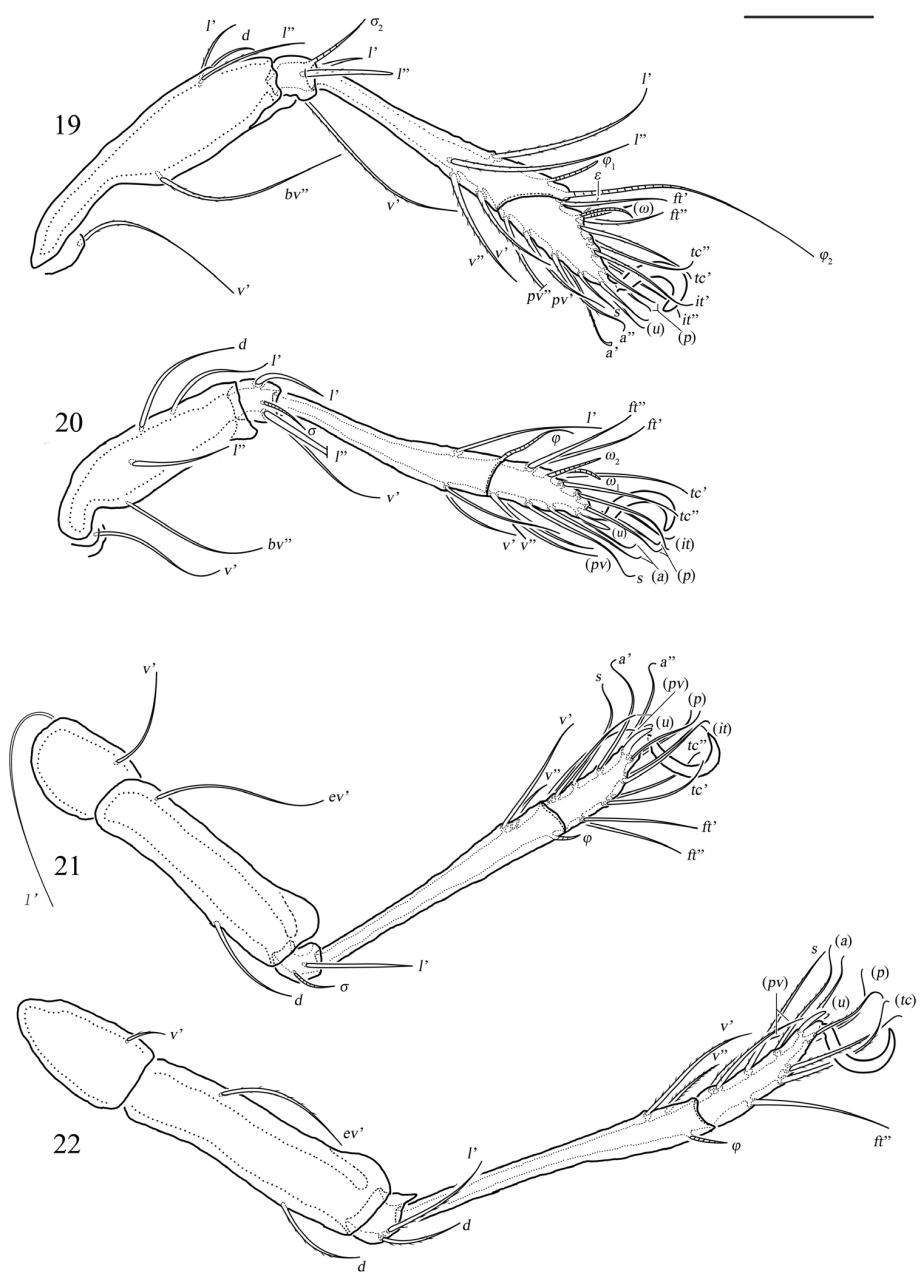
Otocepheus (Acrotocepheus) multigranulatus sp. nov., adult: leg I–IV, right, antiaxial view. Scale bars: 100 µm (**19–22**).

#### Material examined.

Holotype (female, LD-08-10): China, Hunan Province, Yanling County, Taoyuan Dong, 26°17'42"N, 114°1'15"E, 1065 m a.s.l., in soil and debris under trees, 6 July 2008, collected by Dong Liu. Two paratypes (males, LD-08-49): China, Hunan Province, Guidong County, Xinlong Village, 26°4'29"N, 113°46'53"E, 1525 m a.s.l., in soil and debris under trees, 12 July 2008, collected by Dong Liu.

#### Type deposition.

All type specimens are deposited in the collection of IZAS.

#### Etymology.

The specific name “*multigranulatus*” is from Latin for “granulate” and is in reference to the uneven, granular body surface.

#### Remarks.

The new species is similar to O. (A.) lienhardorum Mahunka, 2000 from Malaysia, O. (A.) macrodentatus Hammer, 1981 from Java, and O. (A.) holtmanni Aoki, 1965 from New Guinea in having similar shaped lateral notogastral condyles. However, it differs from O. (A.) lienhardorum by the granulate ventral plate (versus foveolate), notogastral setae nearly same in length (versus setae *p*_1_–*p*_3_ and *h*_3_ long), bothridial seta with a fusiform head (versus markedly developed), and seta *1a* well visible (versus minute or absent). It differs from O. (A.) macrodentatus by the epimeral setal formula 3-1-3-3 (versus 3-1-2-3), normal seta *h*_3_ (versus much smaller and thinner than the other notogasral setae), surface of notogaster densely, and obviously granulate (versus punctate). It differs from O. (A.) holtmanni by the granulate notogastral surface (versus minutely and densely punctured), lyrifissure *ips* located between setae *h*_3_ and *p*_3_ (versus anterior to seta *h*_3_), the largest width of notogaster medially, near level of seta *lp* (versus the largest width rather anteriorly, near level of seta *lm*), vitta marginalis well developed, faint anteriorly (versus vitta marginalis visible only on anterior half of notogaster).

### Otocepheus (Acrotocepheus) occultatus sp. nov.

Taxon classificationAnimaliaSarcoptiformesOtocepheidae

84C7F7DE-33FA-5CAB-B92A-B77BA843282B

http://zoobank.org/16C79F8A-145D-40B4-9204-E2D6DC7124A0

**Figures 23–26.**
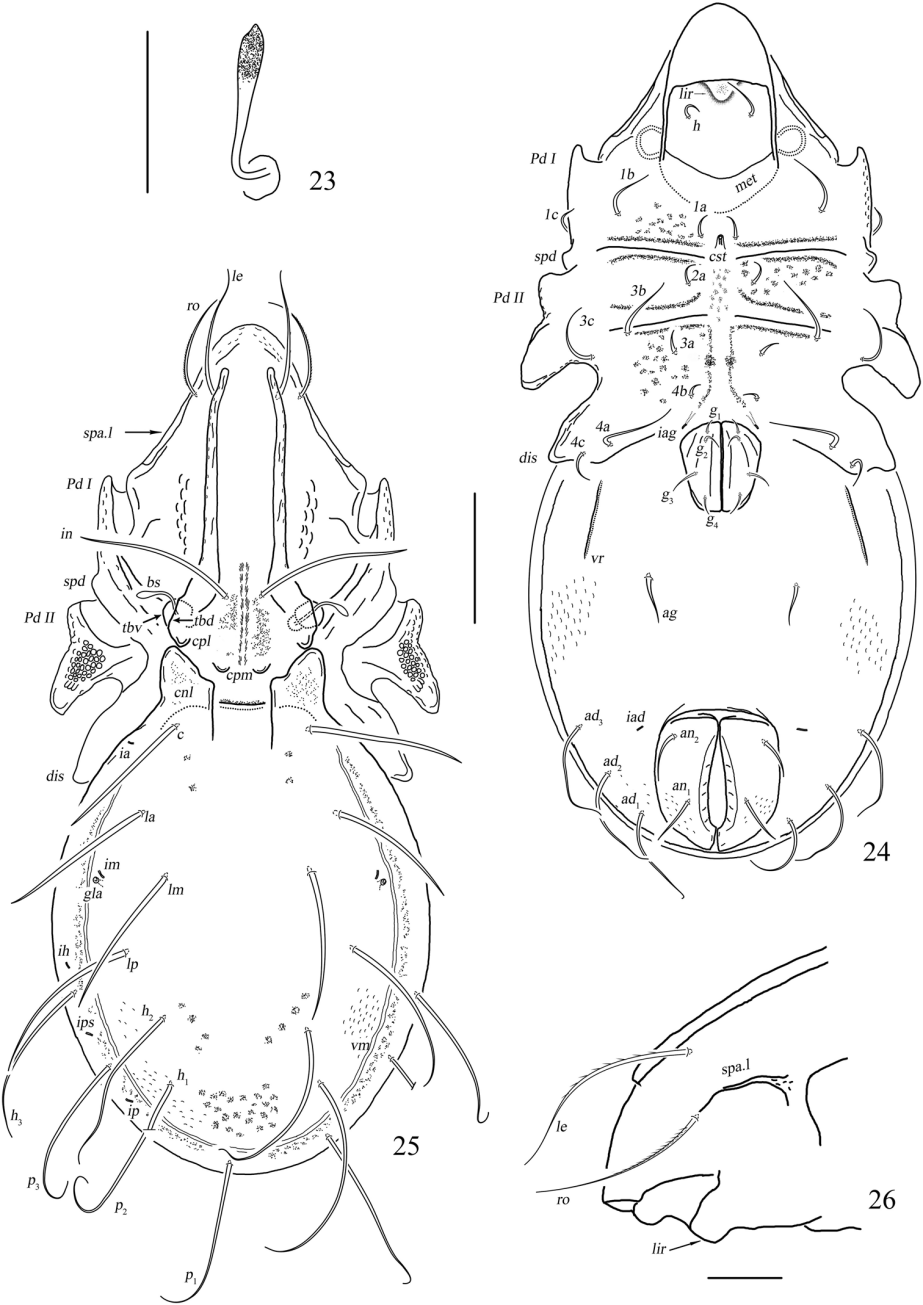
Otocepheus (Acrotocepheus) occultatus sp. nov., adult **23** bothridial seta **24** ventral view (legs not shown) **25** dorsal view **26** lateral lamelliform expansion. Scale bars: 100 µm (**23, 26**), 200 µm (**24, 25**).

**Figure 27.**
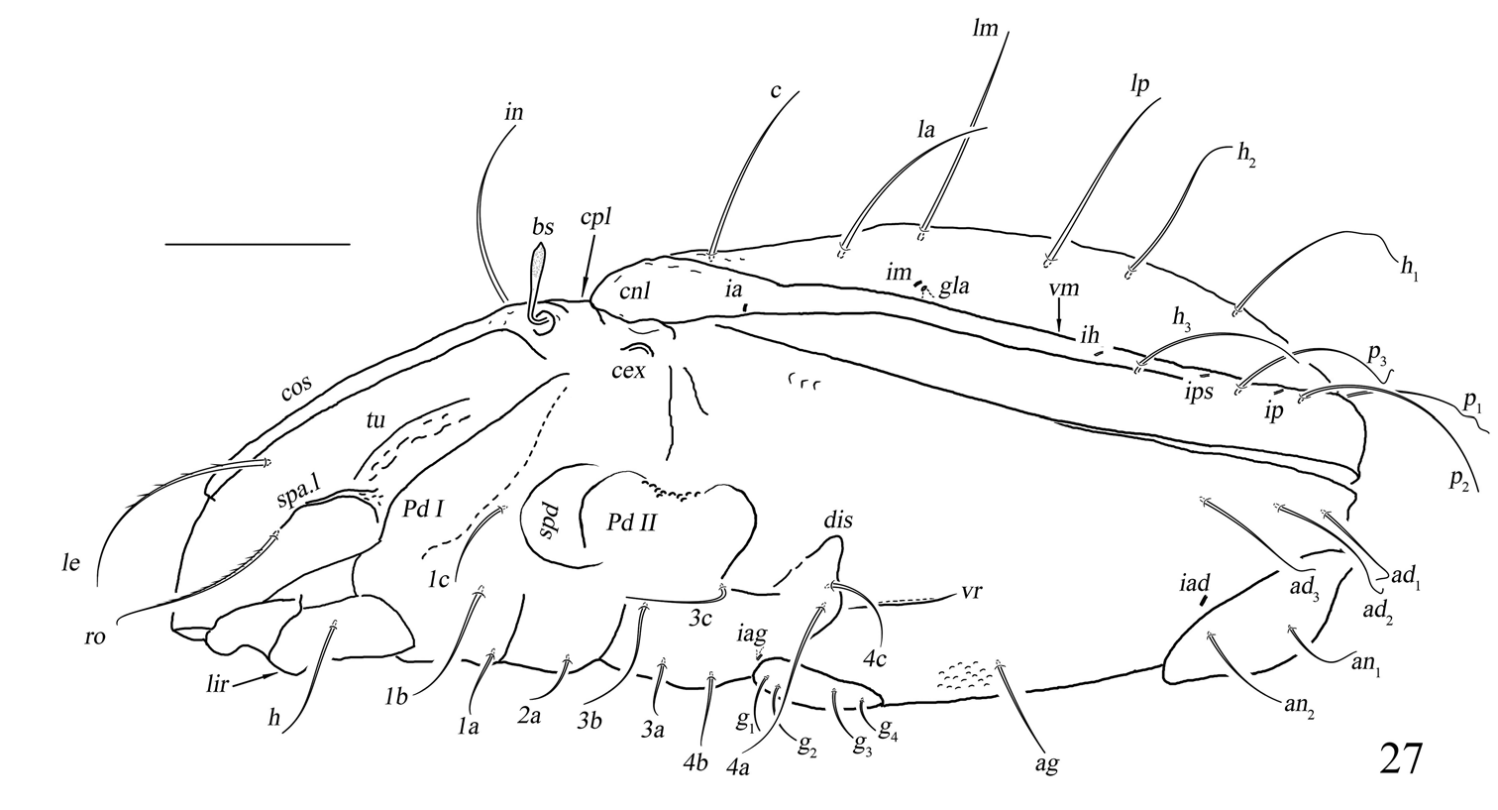
Otocepheus (Acrotocepheus) occultatus sp. nov., adult: lateral view (legs not shown). Scale bar: 200 µm.

**Figures 28–30.**
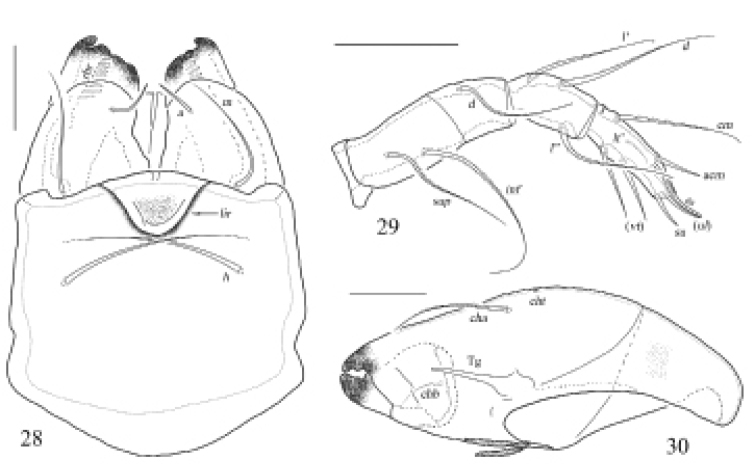
Otocepheus (Acrotocepheus) occultatus sp. nov., adult. **28** subcapitulum, ventral view **29** right palp, abaxial view **30** right chelicera, adaxial view. Scale bars: 50 μm.

Figs 23–26 , 27 , 28–29

#### Diagnosis.

Body size (*n* = 4): 1240–1410 × 560–670. Bothridial seta with a long fusiform head and a short peduncle. Lateral prodorsal condyles on prodorsum small, rounded, lateral prodorsal condyles markedly anterior to medial prodorsal condyles. An extra pair of condyles located posterior to lateral prodorsal condyles, covered by lateral notogastral condyles. Lateral notogastral condyles large, width nearly equal with their mutual distance, tips markedly anterior to medial prodorsal condyles. Anterior median part of mentum with a lower lip ridge.

#### Description.

***Measurements*.** Body length: 1280 (holotype: female), 1240–1410 (three paratypes: one female and two males); notogaster width: 530 (holotype), 560–670 (paratypes). Setae length and mutual distance (holotype): *bs* 85, *in* 220, *le* 235, *ro* 175, *ex* 19; *c*, *la*, *lm*, *lp*, *h*_1_, *h*_2_, *h*_3_, *p*_1_, *p*_2_, *p*_3_ range 150–300; *c*–*c* 200, *la*–*la* 300, *lm*–*lm* 230, *lp*–*lp* 350, *h*_2_–*h*_2_ 225, *h*_1_–*h*_1_ 230.

***Integument*.** Body color light yellow-brownish. Body surface densely foveolate.

***Prodorsum*.** Rostrum rounded. Rostral seta curved inward, densely barbed outside. Lamellar seta inserted behind tip of costula, moderately curved inward, roughened outside. Interlamellar seta barbed, setiform. Bothridial seta with a long fusiform head and a short peduncle. Exobothridial seta short. Costula straight, nearly paralleled. Bothridium opening laterally, dorsal bothridial plate straight, ventral bothridial plate broadly rounded in dorsal view. Lamelliform expansion pointing to bottom of seta *ro.* Two pairs of prodorsal condyles present. Lateral prodorsal condyles small, rounded, markedly anterior to medial prodorsal condyle. Median prodorsal condyles small, rounded, well separated from each other. An extra pair of condyles located posterior to lateral prodorsal condyles, covered by lateral notogastral condyles. Mutual distance between ventral bothridial plates larger than that between lateral prodorsal condyles. Subpedotectum well developed.

***Notogaster*.** L/W of notogaster about 1.3. Surface of notogaster densely punctate. Lateral notogastral condyles large, triangular and rounded distally, inner part with a narrow base, anteromedial margins distinctly excavated, width nearly equal with their mutual distance, tips markedly anterior to medial prodorsal condyles. Medial notogastral condyles absent. Ten pairs of notogastral setae, slightly barbed, setae located posteriorly tend whip-like at tips. All lyrifissures well visible, *ip* located between setae *p*_2_ and *p*_3_, *ips* between setae *h*_3_ and *p*_3_. Opisthonotal gland opening located posterior to lyrifissure *im.* Vitta marginalis distinct.

***Epimeral and lateral podosomal regions*.** Surface punctured. Apodemes II and sejugal well developed, apodeme III invisible. Carina sterinalis short. Epimeral setal formula 3-1-3-3. Seta *4a* inserted between *4b* and *4c*, and closer to *4c*. Postpodosomal ornamentation invisible.

***Anogenital region.*** A pair of longitudinal ridges posterior to epimeral setae *4a*. Genital plates with two or three longitudinal ridges on both sides. Four pairs of genital setae smooth (mutual distances *g*_1_–*g*_1_≈*g*_2_–*g*_2_≈*g*_4_–*g*_4_<*g*_3_–*g*_3_). Aggenital lyrifissure located close and anterolateral to genital aperture. One pair of aggenital, two pairs of anal (mutual distances *an*_1_–*an*_1_<*an*_2_–*an*_2_), and three pairs of adanal setae. Anal setae barbed and blunt at tips, adanal setae barbed and whip-like at tips. Seta *an*_2_ well separated from outer margin of anal plate. Location of adanal setae normal, inside external margin of ventral plate. Setae *ad*_3_–*ad*_3_ below level of anterior margin of anal opening. Adanal lyrifissure located in diagonal position and close to anal aperture.

***Gnathosoma.*** Anterior median part of mentum with a lower lip ridge. Subcapitular setae relatively smooth. Adoral setae and their alveoli absent. Rutellum pantelobasic, with typical dentition and rutellar brush. Chelicera chelate-dentate; with a minute denticle proximal to seta *cha*; *cha* longer than *chb*, both of them setiform, barbed; Trägårdh’s organ narrowly triangular. Palp with usual setal formula: 0–2–1–3–8 (+*ω*); setae of femur to tibia barbed. Tarsus with four short, blunt distal eupathidia–*acm*, *su*, (*ul*); other tarsal setae smooth or with sparse, inconspicuous barbs; solenidion *ω* connected with seta *ul*’, seta *ul*” medioanteriorly. Postpalpal seta erect, smooth.

***Legs*.** Monodactylous. Claw of each leg strong and smooth. Formulae of leg setation and solenidia: I (1-4-3-4-16) [1-2-2], II (1-4-3-3-15) [1-1-2], III (2-3-1-2-14) [1-1-0], IV (1-2-2-2-12) [0-1-0]. Leg seta *u* setiform (L-type) on tarsi I, thorn-like (S-type) on tarsi II–IV. Homology of setae and solenidia indicated in Table 1.

#### Material examined.

Holotype (male, ZLH-12-34): China, Guangxi Province, Wuming County, Daming Mountain, 23°29'42"N, 108°26'17"E, 1223 m a.s.l., in soil and debris under bush, 17 July 2012, collected by Lihao Zheng. Three paratypes (one female and two males, ZLH-12-37): same locality as holotype, 23°28'51"N, 108°27'18"E, 1410 m a.s.l., in soil and debris under tree, 20 July 2012, collected by Lihao Zheng.

#### Type deposition.

All type specimens are deposited in the collection of IZAS.

#### Etymology.

The specific name “*occultatus*” is from Latin for “hiding”, in reference to the extra condyles which are covered by the large lateral notogastral condyles.

#### Remarks.

The new species is most similar to O. (A.) bajau Mahunka, 2000 from Malaysia and O. (A.) consimilis (Balogh, 1970) from Ceylon in having large lateral prodorsal condyles. However, it differs from O. (A.) bajau by the extra condyles on prodorsum covered by lateral notogastral condyles (versus none), bothridial seta with a long fusiform head (versus with a lanceolate head), tarsus I normal (versus with triangular teeth), and it differs from O. (A.) consimilis by the lateral prodorsal condyles markedly anterior to medial prodorsal condyles (versus lateral prodorsal condyles nearly in same line with median prodorsal condyles), seta *an*_2_ well separated from outer margin of anal plate (versus very close to outer margin of anal plate), notogastral setae located posteriorly tend whip-like at tips (versus not), normal location of adanal setae, and inside external margin of ventral plate (versus arising on and parallel with external margin of ventral plate).

### Otocepheus (Acrotocepheus) duplicornutus

Taxon classificationAnimaliaSarcoptiformesOtocepheidae

Aoki, 1965: new record in China

7A6EBD45-479D-5303-AE1D-EDFA7FD7A553

Figs 31–33 , 34–36 , 37–39

Otocepheus (Acrotocepheus) duplicornutus Aoki 1965: 303.
Otocepheus (Acrotocepheus) duplicornutus discrepans (A.): Balogh & Mahunka 1967: 49.

#### Diagnosis.

Body size (*n* = 4): 1150–1400 × 480–600. Body ratio (length/width): 2.3–2.4. Lateral notogastral condyles appears to be double-structured, the outer portion of lateral notogastral condyles triangular anteriorly. Medial notogastral condyles absent. Postpodosomal ornamentation well developed. A pair of longitudinal ridges posterior to epimeral setae *4a* present.

#### Description.

***Measurements*.** Body length: 1150–1400 (four males), notogaster width: 480–600 (four males). Setae length and mutual distance (one male, ZLH-12-73): *bs* 150, *in* 215, *le* 190, *ro* 150, *ex* 35; *c*, *la*, *lm*, *lp*, *h*_1_, *h*_2_, *h*_3_, *p*_1_, *p*_2_, *p*_3_ range 250–300; *c*–*c* 110, *la*–*la* 190, *lm*–*lm* 160, *lp*–*lp* 260, *h*_2_–*h*_2_ 180, *h*_1_–*h*_1_ 190.

***Integument*.** Body color light brown, but genital plates and legs dark brown. Body surface densely foveolate.

***Prodorsum*.** Rostrum rounded. Seta *ro* moderately curved inward, densely barbed outside. Seta *le* removed backward from tip of costula, curved inward, roughened externally. Setae *le* and *in* slightly barbed, with blunt tips. Bothridium opening laterally, dorsal bothridial plate nearly straight, ventral bothridial plate broadly triangular in dorsal view. Bothridial seta with lanceolate head, slightly roughened. Seta *ex* short, setiform. Tutorium developed. Lamelliform expansion pointing to bottom of seta *ro.* Costula well developed. Prodorsal condyles broadly rounded, well separated from each other. Subpedotectum well developed. Extra condyles posterior to lateral prodorsal condyles invisible.

**Figures 31–33.**
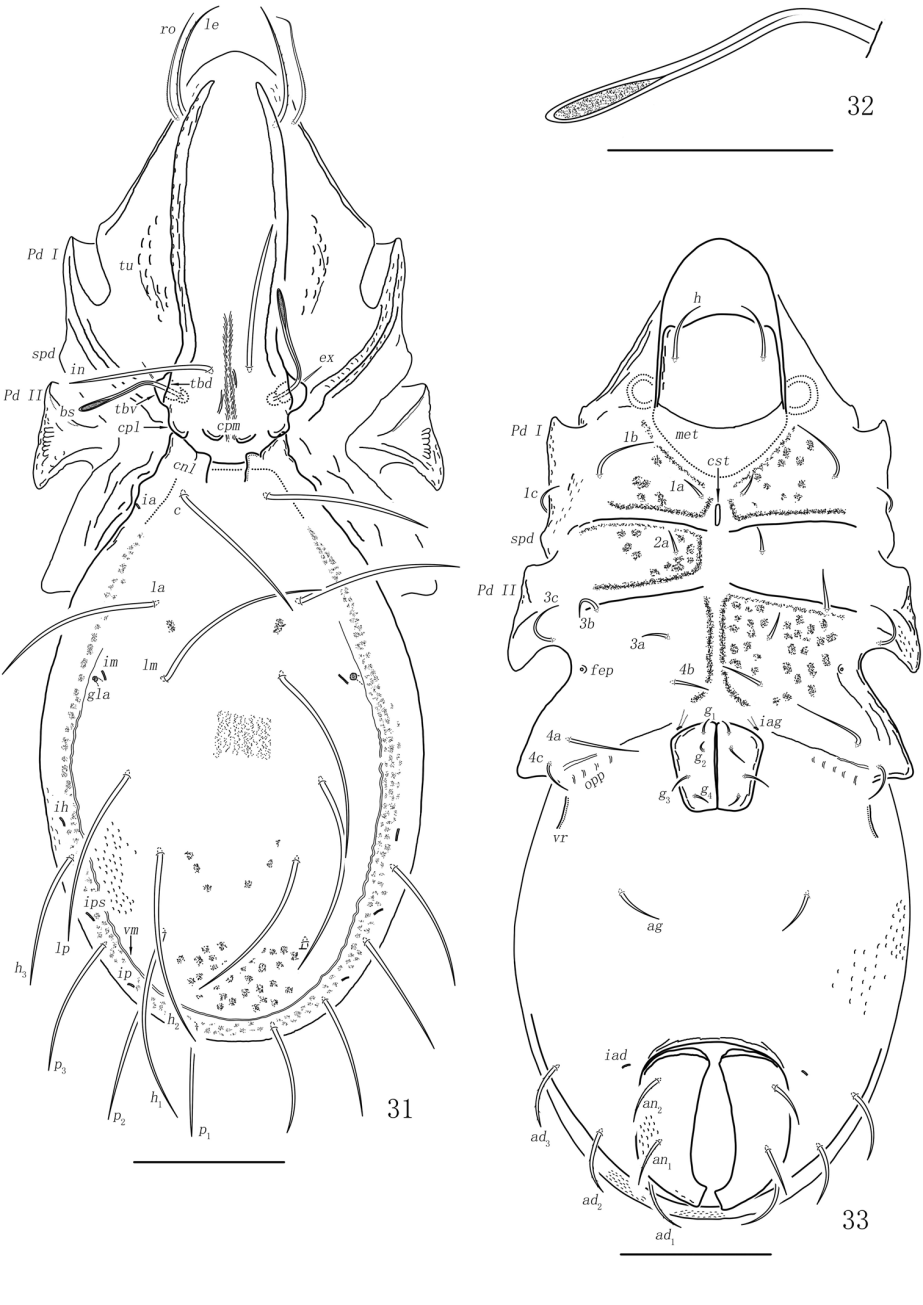
Otocepheus (Acrotocepheus) duplicornutus Aoki, 1965, adult **31** Dorsal view **32** bothridial seta **33** ventral view (legs not shown). Scale bars: 200 µm (**31, 33**), 100 µm (**32**).

***Notogaster*.** Anterior margin of notogater weakly concaved. Lateral notogastral condyles appears to be double-structured, the outer portion of lateral notogastral condyles triangular anteriorly. Medial notogastral condyles absent. Notogaster with ten pairs of setae, setiform, slightly barbed. Lyrifissures distinct, *ip* between setae *p*_2_ and *p*_3_, *ips* between setae *h*_3_ and *p*_3_, *ih* anterior to seta *h*_3_, *im* interiorly to opisthonotal gland opening.

***Epimeral and lateral podosomal regions*.** Apodemes II and sejugal well-developed, apodeme III invisible, epimeral foramen small and rounded. Epimeral setal formula: 3-1-3-3. Setae setiform, *4a* inserted between *4b* and *4c*, and closer to *4c*. Postpodosomal ornamentation well developed.

**Figures 34–36.**
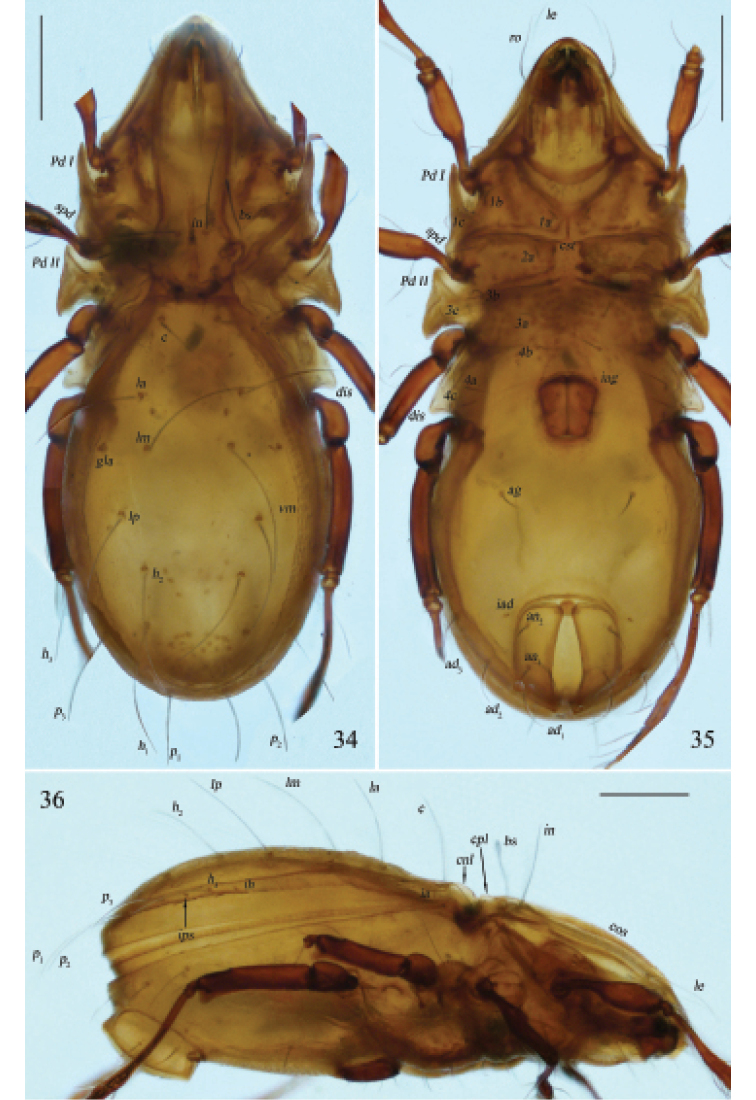
Otocepheus (Acrotocepheus) duplicornutus Aoki, 1965, adult, microscope images **34** dorsal view **35** ventral view **36** lateral view. Scale bars: 200 µm.

***Anogenital region*.** Aggenital lyrifissure located close and anterolateral to genital aperture. A pair of longitudinal ridges posterior to epimeral seta *4a* present. Genital plates smooth. Four pairs of genital setae (mutual distances *g*_1_–*g*_1_≈*g*_2_–*g*_2_≈*g*_4_–*g*_4_<*g*_3_–*g*_3_) and one pair of aggenital setae present, setiform and slightly barbed. Three pairs of adanal and two pairs of anal setae (mutual distance *an*_1_–*an*_1_<*an*_2_–*an*_2_) slightly barbed. Setae *ad*_3_–*ad*_3_ below level of anterior margin of anal opening. Adanal lyrifissure located in diagonal position and close to anal aperture (in some specimens one of the adanal lyrifissures aligned transversely while the other one aligned diagonally), below level of anterior margin of anal opening.

***Gnathosoma*.** Subcapitular setae fistulous, barbed. Adoral setae and their alveoli absent. Rutellum pantelobasic, with typical dentition and rutellar brush. Chelicera chelate-dentate; with a minute denticle proximal to seta *cha*; *cha* longer than *chb*, both of them setiform, barbed; Trägårdh’s organ narrowly triangular. Palp with usual setal formula: 0–2–1–3–8 (+ω); setae of femur to tibia barbed. Tarsus with four short, blunt distal eupathidia – *acm*, *su*, (*ul*); other tarsal setae smooth or with sparse, inconspicuous barbs; solenidion ω connected with seta *ul*’, seta *ul*” medioanteriorly. Postpalpal seta erect, smooth.

**Figures 37–39.**
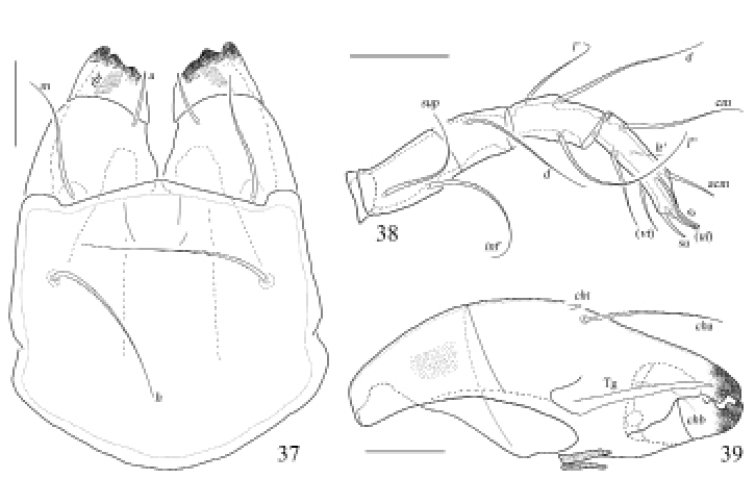
Otocepheus (Acrotocepheus) duplicornutus Aoki, 1965, adult **37** subcapitulum, ventral view **38** right palp, abaxial view **39** left chelicera, adaxial view. Scale bars: 50 μm.

***Legs*.** Claw of each leg strong and smooth. Formulae of leg setation and solenidia: I (1-4-3-4-16) [1-2-2], II (1-4-3-3-15) [1-1-2], III (2-3-1-2-15) [1-1-0], IV (1-2-2-2-12) [0-1-0]. Leg seta *u* setiform (L-type) on tarsi I, thorn-like (S-type) on tarsi II–IV. Homology of setae and solenidia indicated in Table 1.

#### Material examined.

One male (ZLH-12-72): China, Guangxi Province, Longzhou County, 22°25'19"N, 106°58'12"E, 149 m a.s.l., in soil and debris beside deadwood, 3 August 2012; one male (ZLH-12-73): same locality as ZLH-12-72, 22°25'11"N, 106°58'6"E, 154 m a.s.l., in soil and debris beside deadwood, 3 August 2012; one male (ZLH-12-74): same locality as ZLH-12-72, 22°25'7"N, 106°58'2"E, 166 m a.s.l., in soil and debris under liana, 3 August 2012; one male (ZLH-12-77): China, Guangxi Province, Fusui County, 22°27'36"N, 107°53'24"E, 100 m a.s.l., in soil and debris under leaf wood, 8 August 2012. All specimens were collected by Lihao Zheng.

#### Specimen deposition.

Specimens are deposited in the collection of IZAS.

#### Remarks.

The morphological characters of specimens checked in this study are almost coincident with the original description of this species by Aoki (1965), which was based on material collected from Sara Buri, Thailand, except for the following delicate differences: arc degrees of prodorsal condyles (broadly rounded versus semicircular) and alignment of adanal lyrrifissure (located in diagonal position versus generally aligned transversely). Though these characters are relatively constant in our limited specimens, the shape of prodorsal or notogastral condyles, as well as the alignment of adanal lyrrifissure, sometimes vary. So, we temporarily treat these minor differences as normal individual variation.

### Key to known species of Otocepheus (Acrotocepheus) from China

**Table:** 

1	Notogastral setae not longer than distance from nearest one	***O* . (*A* .) *gracilis* Aoki, 1973**
–	Notogastral setae distinctly longer than distance from nearest one	**2**
2	Surface of notogaster with densely small convex granules, lateral notogastral condyles wide, like a lateral view of staircase with two to three steps mainly	***O* . (*A* .) *multigranulatus* sp. nov.**
–	Surface of notogaster densely foveolate, lateral notogastral condyles not as above	**3**
3	Lateral notogastral condyles finger-shaped with wide base, notogastral setae *c*, *la*, *h*_3_, *p*_1_, *p*_3_ setiform and relatively short in length, while the rest flagelliform and distinctly longer in length	***O* . (*A* .) *digitatus* sp. nov.**
–	Lateral notogastral condyles portion triangulate, notogastral setae nearly equal in length	**4**
4	A pair of extra condyles covered by lateral notogastral condyles , anterior median part of mentum with a lower lip ridge, notogastral setae located posteriorly tend whip-like at tips	***O* . (*A* .) *occultatus* sp. nov.**
–	No extra condyles under lateral notogastral condyles present, anterior median part of mentum relatively flat, all notogastral setae setiform, without whip-like tipes	***O* . (*A* .) *duplicornutus* Aoki, 1965**

## Supplementary Material

XML Treatment for Otocepheus (Acrotocepheus) digitatus

XML Treatment for Otocepheus (Acrotocepheus) multigranulatus

XML Treatment for Otocepheus (Acrotocepheus) occultatus

XML Treatment for Otocepheus (Acrotocepheus) duplicornutus
